# Circular RNA CDR1as Mediated by Human Antigen R (HuR) Promotes Gastric Cancer Growth via miR-299-3p/TGIF1 Axis

**DOI:** 10.3390/cancers15235556

**Published:** 2023-11-23

**Authors:** Rong Li, Xuejing Xu, Shuo Gao, Junyi Wang, Jie Hou, Zhenfan Xie, Lan Luo, Han Shen, Wenrong Xu, Jiajia Jiang

**Affiliations:** 1 Department of Laboratory Medicine, Nanjing Drum Tower Hospital, The Affiliated Hospital of Nanjing University Medical School, Nanjing University, 321 Zhongshan Road, Nanjing 210008, China; ellenli_0422@163.com (R.L.); xxuejing@hotmail.com (X.X.); shuoshuo65@163.com (S.G.); shenhan10366@sina.com (H.S.); 2 Jiangsu Key Laboratory of Medical Science and Laboratory Medicine, School of Medicine, Jiangsu University, 301 Xuefu Road, Zhenjiang 212013, China; ujshj2311@163.com (J.H.); didilouis@163.com (Z.X.); luo10918@163.com (L.L.); 3 Centre of Clinical Laboratory, The First Affiliated Hospital of Soochow University, 899 Pinghai Road, Suzhou 215006, China; wjyandjay@sina.com; 4 Aoyang Institute of Cancer, Affiliated Aoyang Hospital of Jiangsu University, 279 Jingang Road, Suzhou 215600, China

**Keywords:** CDR1as, gastric cancer, miR-299-3p, TGIF1, HuR

## Abstract

**Simple Summary:**

Gastric cancer (GC) is one of the most common malignancies with limited therapeutic targets available. CDR1as, an endogenous circular RNA with a closed loop structure, has been reported to be a crucial regulator and promising biomarker in various tumors. However, whether and how CDR1as participates in GC progression remains not well characterized. In this study, we explored the biological roles, the downstream molecular mechanism and the upstream regulator of CDR1as in GC. We found that CDR1as mediated by human antigen R (HuR) promotes GC growth through the miR-299-3p/TGIF1 axis, which provides new insights into GC pathogenesis and brings a new potential target for clinical GC therapy.

**Abstract:**

**Background:** Gastric cancer (GC) remains a common malignancy worldwide with a limited understanding of the disease mechanisms. A novel circular RNA CDR1as has been recently reported to be a crucial regulator of human cancer. However, its biological role and mechanism in the GC growth are still far from clear. **Methods:** Small interfering RNAs (siRNAs), lentivirus or plasmid vectors were applied for gene manipulation. The CDR1as effects on the GC growth were evaluated in CCK8 and colony formation assays, a flow cytometry analysis and mouse xenograft tumor models. A bioinformatics analysis combined with RNA immunoprecipitation (RIP), RNA pull-down assays, dual-luciferase reporter gene assays, Western blot, reverse transcription–quantitative polymerase chain reaction (RT-qPCR) and functional rescue experiments were used to identify the CDR1as target miRNA, the downstream target gene and its interaction with human antigen R (HuR). **Results:** The CDR1as overexpression promoted the GC growth in vitro and in vivo and reduced the apoptotic rate of GC cells. Its knockdown inhibited the GC cell proliferation and viability and increased the cell apoptotic rate. Proliferation-related proteins PCNA and Cyclin D1 and apoptosis-related proteins Bax, Bcl-2, Caspase-3 and Caspase-9 were regulated. Mechanically, the cytoplasmic CDR1as acted as a miR-299-3p sponge to relieve its suppressive effects on the GC cell growth. Oncogenic TGIF1 was a miR-299-3p downstream target gene that mediated the promotive effects of CDR1as and regulated the PCNA and Bax levels. HuR interacted with CDR1as via the RRM2 domain and positively regulated the CDR1as level and its oncogenic role as well as downstream target TGIF1. **Conclusions:** CDR1as promotes the GC growth through the HuR/CDR1as/miR-299-3p/TGIF1 axis and could be used as a new therapeutic target for GC.

## 1. Introduction

Gastric cancer (GC) ranks as the fifth most common malignancy and the third leading cause of cancer-related death worldwide [1]. *Helicobacter pylori* infection, environmental factors (diet and exogenous chemicals) and genetic abnormalities are the main risk factors of gastric tumorigenesis [2]. Unlimited cell growth is an important feature of GC and is closely associated with tumor metastasis and other malignant progression. However, the specific molecular mechanisms of the GC growth remain far from clear. Traditional surgery resection, chemotherapy and radiation therapy are still unsatisfactory due to severe systemic cytotoxicity and drug resistance [3,4]. Thus, exploring the underlying mechanisms of the GC progression and developing new therapeutic targets are important for improving the prognosis of GC patients.

Circular RNAs (circRNAs), a novel type of RNAs with a covalently closed loop structure, are becoming a new hotspot in the field of cancer. They are generated from exons or introns of pre-mRNAs via a back-splicing process, which ligates a downstream splice donor site to an upstream splice acceptor site [5]. With the advancement of high-throughput RNA deep sequencing technology and bioinformatics, large amounts of circRNAs have been identified in various tumors [6]. Due to their high stability and abundance, evolutionary conservation and tissue- and developmental stage-specific expression patterns, circRNAs are considered to be promising tumor biomarkers [7,8,9]. Moreover, many aberrantly expressed circRNAs participate in multiple processes of tumor pathogenesis by sponging miRNAs, interacting with proteins or encoding functional peptides [10]. For example, hsa_circ_0023404 enhances cervical cancer (CC) metastasis and chemoresistance by sponging miR-5047 to upregulate the VEGFA level [11]. CircACTN4 promotes the growth and metastasis of breast cancer (BRC) by competitively binding to FUBP1 with FIR to transcriptionally activate MYC [12]. In addition, several circRNAs such as circβ-catenin, circGprc5a and circPPP1R12A can encode proteins to promote liver cancer, bladder cancer and colon cancer progression [13]. Thus, circRNAs are crucial tumor regulators and proposed to be potential therapeutic targets for tumor treatment. In GC, a growing number of circRNAs have been reported to regulate tumor proliferation, apoptosis, migration and invasion through distinct mechanisms [14]. For instance, circTHBS1 promotes the growth and metastasis of GC by sponging miR-204-5p to enhance the INHBA expression [15]. CircNFATC3 promotes GC proliferation by binding to IGF2BP3 and restricting its ubiquitination to enhance the CCND1 mRNA stability [16]. Further research of GC-related circRNAs would provide a new understanding of tumor pathogenesis and targets for the GC therapy.

CDR1as (ciRS-7) is one of the most extensively investigated circRNAs. It is transcribed from the CDR1 gene but driven by the promoter of LINC00632 and cleaved by miR-671 in an Ago2 slicer-dependent manner [17,18]. CDR1as has been reported to play crucial roles in multiple physiological and pathological processes including stem cell differentiation [19], insulin secretion [20], neuropsychiatric disorders [21], osteoarthritis [22] and especially tumor [23]. It promotes the growth and metastasis of non-small cell lung cancer (NSCLC) [24], osteosarcoma (OS) [25], esophageal squamous cell carcinoma [26] and pancreatic ductal adenocarcinoma [27] while suppressing ovarian cancer (OC) [28]. Most studies focus on the role of CDR1as as a miR-7 sponge since it harbors over 70 conserved binding sites for miR-7 and could suppress its functions [29]. Our previous study indicated that CDR1as is a potential regulator and promising biomarker for GC [30]. However, its biological roles and mechanisms of action in the GC progression are not well characterized. 

**Figure 1:**
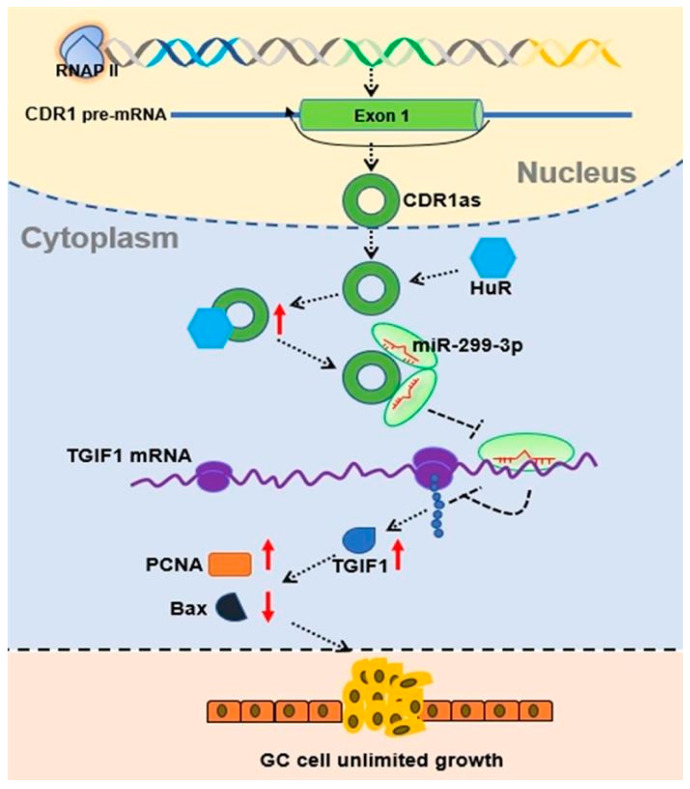
Schematic outlining the mechanism of CDR1as in GC growth. HuR interacts with CDR1as and increases its expression level to attenuate miR-299-3p suppression of TGIF1 mRNA, which in turn upregulates (red up arrow) TGIF1 and downstream PCNA while downregulating (red down arrow) Bax to promote GC growth.

In this study, we observed the promotive effects of CDR1as on the GC growth and found a new CDR1as/miR-299-3p/TGIF1 interaction network in GC. Moreover, we identified the human antigen R (HuR) as the regulator of CDR1as (Figure 1). Our findings elucidate a novel underlying mechanism of the GC growth and provide a potential therapeutic target for the GC treatment in the future. 

## 2. Materials and Methods

### 2.1. Cell Lines and Culture Conditions

The human GC cell lines HGC-27, SGC-7901, BGC-823, MGC-803, AGS and MKN-45 were purchased from the Cell Bank of the Chinese Academy of Sciences (Shanghai, China). MKN-45 and MGC-803 cells were cultured in high-glucose DMEM (Gibco, Grand Island, NY, USA) containing 10% fetal bovine serum (FBS; Bovogen, Keilor East, VIC, Australia). HGC-27 and SGC-7901 cells were maintained in RPMI-1640 (Bioind, Beit Haemek, Israel) with 10% FBS. AGS cells were propagated in DMEM/F-12 Medium (Bioind, Beit Haemek, Israel) supplemented with 10% FBS. All cells were grown at 37 °C in a water-saturated incubator with a 5% CO_2_ atmosphere. 

### 2.2. Nuclear–Cytoplasmic Fractionation, RNA Extraction and Reverse Transcription–Quantitative Polymerase Chain Reaction (RT-qPCR)

Nuclear and cytoplasmic RNA fractionation of cells was performed with RNeasy Mini Kit (Qiagen, Hilden, Germany). GC tissues and adjacent normal tissues of patients were collected as previously reported [30]. The remaining nuclear RNA and total RNA from cells or tissues were isolated with TRIzol reagent (Invitrogen, Waltham, MA, USA) according to the manufacturer’s protocol. The RNA concentration and purity were determined by NanoDrop 2000 (Thermo Fisher Scientific, Waltham, MA, USA). RT-qPCR for mRNAs and circRNAs was conducted with HiScript 1st Strand cDNA Synthesis Kit and AceQ qPCR SYBR Green Master Mix (Vazyme, Nanjing, China). MiRNAs were transcribed with miScript II RT Kit and detected with miScript SYBR Green PCR Kit (Qiagen, Hilden, Germany). The ABI StepOnePlus Real-Time PCR System (Thermo Fisher Scientific, Waltham, MA, USA) was used for all quantitative analyses. β-Actin was used to normalize mRNAs and circRNAs, while U6 was used for miRNAs normalization. The expression levels were presented by the −△Ct method, and the relative expression levels were calculated by the 2^−△△Ct^ method. PCR products were separated by 1.5% agarose gels and examined by UV irradiation. The primer sequences for circRNAs and mRNAs are listed in Supplementary File S1, Table S1. The primers for miRNAs and U6 were purchased from Qiagen (Germany).

### 2.3. CDR1as Stably Overexpressing GC Cells 

CDR1as-overexpressing cell lines were constructed via a lentivirus expression vector system (Hanbio Biotechnology, Shanghai, China). MKN-45, AGS, BGC-823 and SGC-7901 cells were transduced with a green fluorescent protein (GFP) labeled lentivirus vector HBLV-CDR1as-GFP-PURO or HBLV-GFP-PURO (MOI = 30) and selected with 2 μg/mL puromycin (Invitrogen, USA) for 15 days. The fluorescence intensity of GFP was evaluated by fluorescence microscope, and the efficiency of CDR1as overexpression was determined by RT-qPCR analysis.

### 2.4. Transfection, Oligonucleotides and Plasmids

Two different small interfering RNAs (siRNAs) targeting CDR1as (back-spliced junction site), TGIF1 or HuR were designed and synthesized for their knockdown (GenePharma, Shanghai, China). The coding sequence (CDS) of TGIF1 or HuR was synthesized and cloned into pcDNA3.1(+) plasmids to construct their overexpression vectors (GenePharma, China). The wild-type and deletion mutants of HuR vectors were designed by GenScript (Zhenjiang, China). MiRNA mimics (miR-876-5p, miR-3167, miR-299-3p, miR-203a) were purchased (GenePharma, China) for miRNA overexpression. Cells were seeded and cultured in 6-well plates (2 × 10^5^/well) or 10 cm dishes (6 × 10^5^/dish) overnight before these oligonucleotides or vectors were transfected into cells with Lipofectamine 2000 (Invitrogen, USA) in serum-free medium at a concentration of 25 nM for 6 h and then changed with complete medium. Cells were harvested 48 h after transfection for RNA analysis and functional experiments or 72 h for protein analysis. The sequences of oligonucleotides were listed in Supplementary File S1, Table S2.

### 2.5. Flow Cytometry Apoptosis Assay

Cell apoptosis assays were determined by flow cytometry using Annexin V/propidium iodide (PI) apoptosis detection kit (Vazyme, Nanjing, China). Cells were harvested using trypsin without EDTA and resuspended in 1× binding buffer to a concentration of 1 × 10^6^ cells/mL. Then, 5 μL of FITC Annexin V and 1 μL of 100 μg/mL PI solution were added to each 100 μL of cell suspension and incubated at room temperature for 15 min before 400 μL of 1× binding buffer was added. The apoptotic cells were analyzed with CytoFLEX (Beckman, Brea, CA, USA) and quantified with CytExpert 2.3 (Beckman, USA). 

### 2.6. CircRNA Fluorescence In Situ Hybridization (FISH)

RNA FISH for CDR1as was performed using a FISH kit (GenePharma, China). Cells (1 × 10^4^/well) were grown on round coverslips in a 24-well plate overnight. After fixed with 100% ethanol, permeabilized with 0.1% triton X-100, incubated with 2× saline sodium citrate (SCC) and dehydrated in ethanol, cells were hybridized with Cy3-labeled RNA probes at 37 °C overnight and counterstained with Hoechst 33342 (Sigma, Livonia, MI, USA) for 10 min at room temperature. Laser scanning confocal microscopy (Nikon, Tokyo, Japan) was used to visualize and obtain images. The probe sequence was 5′-GGTGCCATCGGAAACCCTGGATATTGCAGACACTGGAAGA-3′. 

### 2.7. Dual-Luciferase Reporter Gene Assay

The recombinant dual-luciferase reporter plasmids were synthesized by GenScript (China). The pmirGLO-CDR1as plasmid was constructed by inserting full-length CDR1as cDNA into vector pmirGLO (Promega, Madison, WI, USA) at 5′ Pmel and 3′ Nhel sites. The pmirGLO-TGIF1WT and pmirGLO-TGIF1Mut vectors were synthesized by inserting the 3′-UTR sequence of TGIF1 or its mutant binding sequence for miR-299-3p into vector pmirGLO at 5′ Nhel and 3′ Xbal sites. GC cells (4 × 10^4^/well) were seeded in 24-well plates overnight before 400 ng luciferase reporter vector and 40 nM miRNA mimics were co-transfected. Cells were harvested after 24 h and analyzed for firefly and Renilla luciferase activity using Dual-Glo Luciferase Assay Kit (Promega, USA) and GloMax 20/20 Luminometer (Promega, USA).

### 2.8. RNA Immunoprecipitation (RIP) Assay

RIP assays were performed with Magna RIP RNA-Binding Protein Immunoprecipitation Kit (Millipore, Burlington, MA, USA) following the manufacturer’s instructions. In brief, 6 × 10^7^ cells were lysed in 300 μL complete RNA lysis buffer. Each 100 μL cell lysate was added to 900 μL RIP immunoprecipitation buffer containing magnetic beads conjugated with 5 μg antibodies and then rotated overnight at 4 °C. After incubation with proteinase K buffer at 55 °C for 30 min, the immunoprecipitated RNA was purified and analyzed with RT-qPCR. 

### 2.9. CCK8 Assay

Cell viability was evaluated using the CCK-8 Cell Counting Kit (Vazyme, Nanjing, China). Cells (4 × 10^3^/well) in 100 μL complete medium were cultured in triplicate 96-well plates for 1–5 days. Then, 10 μL CCK-8 solution was added to each well and incubated at 37 °C for 2 h. The absorbance at 450 nm was measured with an automatic microplate reader (BioTEK, Shoreline, WA, USA).

### 2.10. Colony Formation Assay

Cell proliferation ability was assessed by colony formation assay. Cells (2 × 10^3^/well) were seeded in 6-well plates and cultured for 10–14 days. The medium was changed at an interval of 2–3 days. Colonies were fixed with 4% paraformaldehyde and stained with 0.1% crystal violet.

### 2.11. Western Blot

Western blot was performed with the standard protocol. Cells were harvested and lysed in RIPA buffer supplemented with proteinase inhibitors (Invitrogen, USA) on ice. Equal amounts of proteins were separated by 12% SDS-PAGE gel. After electrophoresis, separated proteins were transferred onto a PVDF membrane (Millipore, USA), blocked in 5% (*w*/*v*) nonfat milk and then incubated with primary antibodies at 4 °C overnight. The sources of primary antibodies were as follows: rabbit anti-β-actin antibody (1:5000; ABclonal, Woburn, MA, USA), rabbit anti-Bax antibody (1:1000; Cell Signaling, Danvers, MA, USA), mouse anti-Bcl-2 antibody (1:200; Cell Signaling, USA), rabbit anti-actived-Caspase-3 antibody (1:500; Bioworld, Bloomingto, MN, USA), mouse anti-Caspase-9 antibody (1:600; Cell Signaling, USA), rabbit anti-PCNA antibody (1:800, Bioworld, USA), rabbit anti-Cyclin D1 antibody (1:500; Bioworld, USA), mouse anti-TGIF1 antibody (1:800; Santa Cruz, CA, USA), rabbit anti-HuR antibody (1:800, ABclonal, Woburn, MA, USA). After incubation with goat anti-rabbit lgG secondary antibody (1:2000; Invitrogen, USA) and goat anti-mouse lgG secondary antibody (1:1000; Invitrogen, USA), the protein band signals were visualized with the enhanced chemiluminescence system (ImageQuant LAS4000mini, GE, Shinjuku, Japan). The densitometry readings of bands were analyzed by Image J software (https://imagej.net/ij/ij/ (accessed on 1 January 2020)) and normalized to β-actin expression. 

### 2.12. Tumor Model

Male BALB/c nu/nu mice (Cavens, Changzhou, China) aged 4–6 weeks were randomly divided into two groups (*n* = 4) and received subcutaneous injections of either CDR1as-overexpressing MKN-45 cells or corresponding negative control cells (5 × 10^6^ cells in 200 μL PBS per mouse). After two weeks, all mice were sacrificed. All experimental procedures involving animals were in accordance with the Guide for the Care and Use of Laboratory Animals and approved by the Animal Use Ethics Committee of Jiangsu University (2012258).

### 2.13. Hematoxylin–Eosin (HE) Staining and Immunohistochemistry (IHC)

The subcutaneous tumors from nude mice were fixed with 4% paraformaldehyde and made into paraffin-embedded tissue sections. For HE staining, sections were deparaffinized in xylene, rehydrated through graded ethanol and stained with hematoxylin–eosin. For IHC staining, a streptavidin–biotin complex (SABC) kit (Boster, Wuhan, China) was used. After deparaffinization and rehydration, sections were performed heat-induced antigen retrieval in citrate buffer (10 mM, pH 6.0) and exposed to 3% hydrogenous peroxidase for 10 min to suppress endogenous peroxidase activity. Then, slides were blocked with 5% bovine serum albumin (BSA), incubated with primary antibody at 4 °C overnight, secondary antibody at 37 °C for 20 min and SABC at 37 °C for 30 min and finally stained with diaminobenzidine (DAB) and hematoxylin for microscopic observation. The primary antibody was rabbit anti-Bax antibody (1:100; Cell Signaling, USA).

### 2.14. RNA Pull-Down Assay

Biotin-labeled sense and antisense probes for CDR1as were synthesized by GenePharma (China). Pierce Magnetic RNA-Protein Pull-Down Kit (Thermo Fisher Scientific, USA) was used for CDR1as pull-down assays. CDR1as-overexpressing MKN-45 cells were seeded in 10 cm dishes. Cells were collected and lysed in 100 μL lysis buffer and then incubated for 1 h at 4 °C with 50 pmol probes which were ligated with 50 μL streptavidin agarose magnetic beads for 30 min at room temperature. After washing and elution, the retrieved protein was detected by Western blot. The sequences of antisense and sense probes were as follows: antisense 5′-GGTGCCATCGGAAACCCTGGATATTGCAGACACTGGAAGA-3′-biotin; sense 5′-TCTTCCAGTGTCTGCAATATCCAGGGTTTCCGATGGCACC-3′-biotin.

### 2.15. Bioinformatics Prediction of Protein–RNA Docking

The secondary structure of CDR1as was provided by the RNAfold WebServer (http://rna.tbi.univie.ac.at/cgi-bin/RNAWebSuite/RNAfold.cgi (accessed on 6 April 2023)) with complete RNA sequence obtained from circBase database (http://www.circbase.org (accessed on 6 April 2023)) [31,32]. The RNAComposer (http://rnacomposer.ibch.poznan.pl (accessed on 6 April 2023)) was used to model RNA 3D structure (up to 500 nucleotides) [33]. The protein sequence of HuR was obtained from the UniProt database (http://www.uniprot.org (accessed on 6 April 2023)) with accession number Q15717, and the protein 3D structure was modeled by SWISS-MODEL (https://swissmodel.expasy.org (accessed on 6 April 2023)) based on the alignment coverage and confidence level [34,35]. The CDR1as-HuR docking prediction was performed on the HDOCK server (http://hdock.phys.hust.edu.cn/ (accessed on 7 April 2023)), and the optimal model was selected based on the provided docking score and predicted binding sites [36]. UCSF ChimeraX version 1.2.5 software was used for 3D structure visualization [37]. 

### 2.16. Statistical Analysis

All statistical analyses were performed using SPSS 21.0 (IBM, Armonk, NY, USA) and GraphPad Prism version 5.0 software (LaJolla, CA, USA). Differences between two experimental groups were assessed by Student’s *t*-test or Mann–Whitney U test. Statistical comparisons for more than three groups were analyzed by one-way ANOVA with post hoc Bonferroni test or Welch’s ANOVA with Games–Howell test. Shapiro–Wilk test was used to evaluate the normality of data distribution, and Levene’s test was performed to verify the homogeneity of variance. All experiments were performed in biological triplicate. Data are presented as the mean ± SD or as median and IQR. For all results, *p* < 0.05 was considered statistically significant.

## 3. Results

### 3.1. CDR1as Promotes GC Growth In Vitro and In Vivo

**Figure 2:**
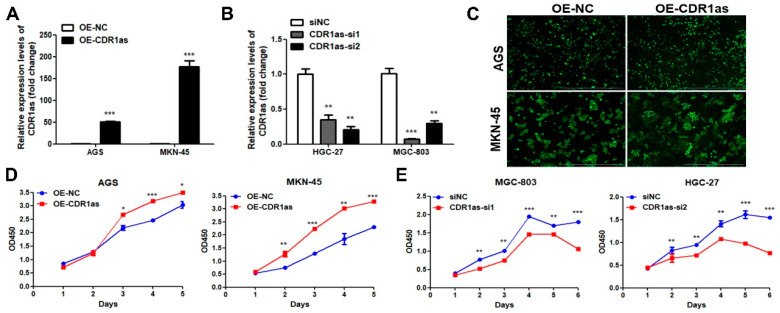

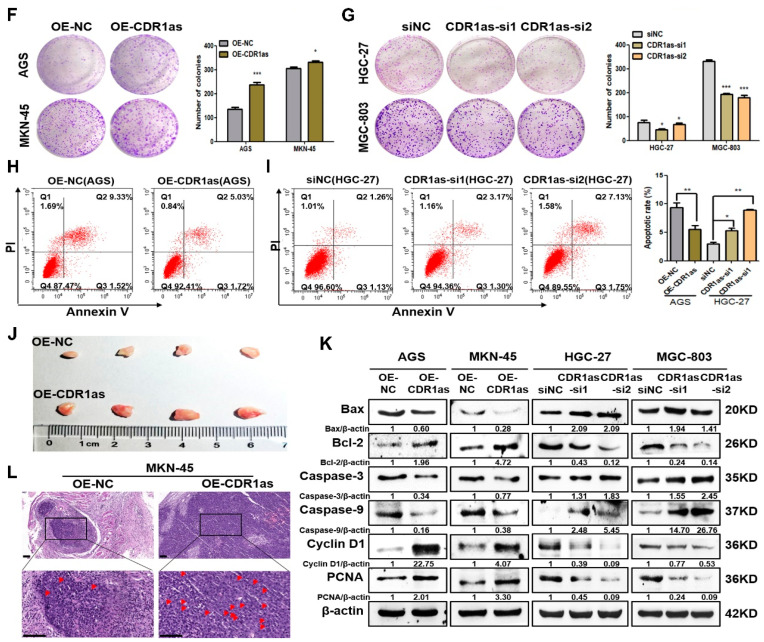
CDR1as promoting GC growth in vitro and in vivo. (**A**,**B**) RT-qPCR analysis of CDR1as level in GC cells after its overexpression and knockdown. (**C**) GFP intensities in CDR1as-overexpressing GC cells and corresponding control cells; the scale bar indicates 1000 μm. (**D**,**E**) Viability of GC cells after CDR1as overexpression and knockdown evaluated by CCK8 assays. (**F**,**G**) Proliferation ability of GC cells after CDR1as overexpression and knockdown assessed with colony formation assays; the bar graphs show the quantitative comparisons of colony numbers. (**H**,**I**) Flow cytometry analysis for the rate of apoptotic cells after CDR1as overexpression and knockdown; the right bar graph shows the quantitative comparisons of apoptotic rate of GC cells. (**J**) Macroscopic appearance and size of dissected tumors. (**K**) Western blot analysis of proliferation- and apoptosis-related proteins after CDR1as overexpression and knockdown in GC cells. (**L**) HE staining of subcutaneous tumor tissues in OE-NC and OE-CDR1as groups; red arrows indicate cells in division (* *p* < 0.05, ** *p* < 0.01, *** *p* < 0.001). The original western blot of Figure 2K is in Supplementary File S3.

To investigate the biological effects of CDR1as on the GC growth, we conducted gain-of-function and loss-of-function assays. We first constructed stable CDR1as-overexpressing GC cell lines via lentivirus-mediated transfection. The transduced GC cells (AGS, MKN-45, SGC-7901 and BGC-823) all showed high intensities of green fluorescence (Figure 2C; Supplementary File S2, Figure S1A). The RT-qPCR results suggested the successful construction of the CDR1as-overexpressing GC cell lines (Figure 2A; Supplementary File S2, Figure S1E). We also knocked down the CDR1as level in two GC cell lines (HGC-27 and MGC-803). Two siRNAs targeting the back-spliced junction sites of CDR1as were transfected, and a nonspecific siRNA was used as the negative control. The knockdown efficiency was confirmed by RT-qPCR (Figure 2B). The CCK8 assays revealed that the CDR1as-overexpressing cells (especially MKN-45 cells) had higher cell viability compared to the control cells (Figure 2D; Supplementary File S2, Figure S1D). The CDR1as knockdown with two siRNAs significantly decreased the viability of the GC cells (Figure 2E; Supplementary File S2, Figure S1C). The colony formation assays also indicated that the CDR1as-overexpressing GC cells had enhanced proliferation ability (Figure 2F; Supplementary File S2, Figure S1B). When CDR1as was knocked down, the GC cell growth was inhibited (Figure 2G). Moreover, the CDR1as overexpression markedly reduced the apoptotic rate of the GC cells, while its knockdown induced cell apoptosis (Figure 2H,I). We then detected the expression levels of proliferation- and apoptosis-related proteins. The proliferation-related proteins PCNA and Cyclin D1 were significantly increased in the CDR1as-overexpressing GC cells. Meanwhile, the expression levels of Bax, caspase-3 and caspase-9 were downregulated, while that of Bcl-2 was upregulated with the CDR1as overexpression. The CDR1as knockdown exhibited the opposite effects (Figure 2K). 

Additionally, we established subcutaneous xenograft tumor models. The CDR1as-overexpressing MKN-45 cells (OE-CDR1as) and the corresponding negative control cells (OE-NC) were injected into the right armpit of nude mice. Increased tumor sizes were observed in the OE-CDR1as group compared with those in the OE-NC group (Figure 2J). More cell division could be found in OE-CDR1as tumors through HE staining (Figure 2L), suggesting that CDR1as promoted the GC cell proliferation in vivo. IHC staining also confirmed that the expression of apoptosis-inducing protein Bax was significantly decreased in the OE-CDR1as group (Supplementary File S2, Figure S1F). Collectively, these results suggested that CDR1as promotes the GC growth in vitro and in vivo.

### 3.2. CDR1as Acts as a miRNA Sponge for miR-299-3p

**Figure 3:**
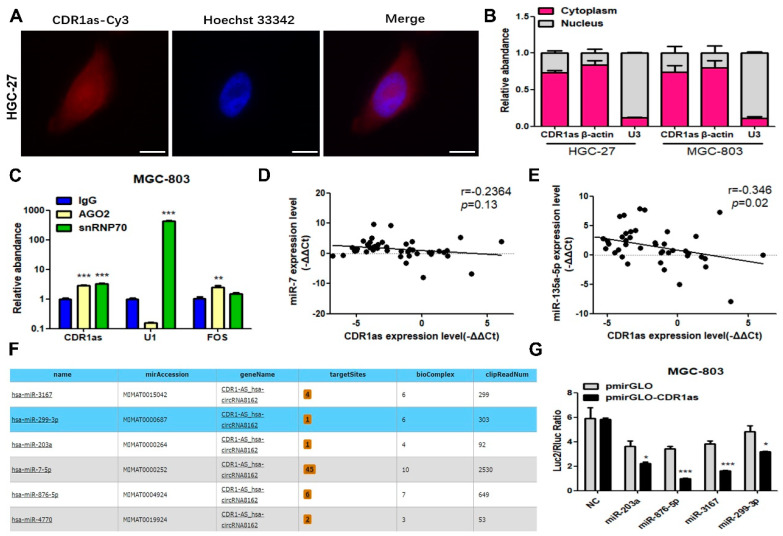

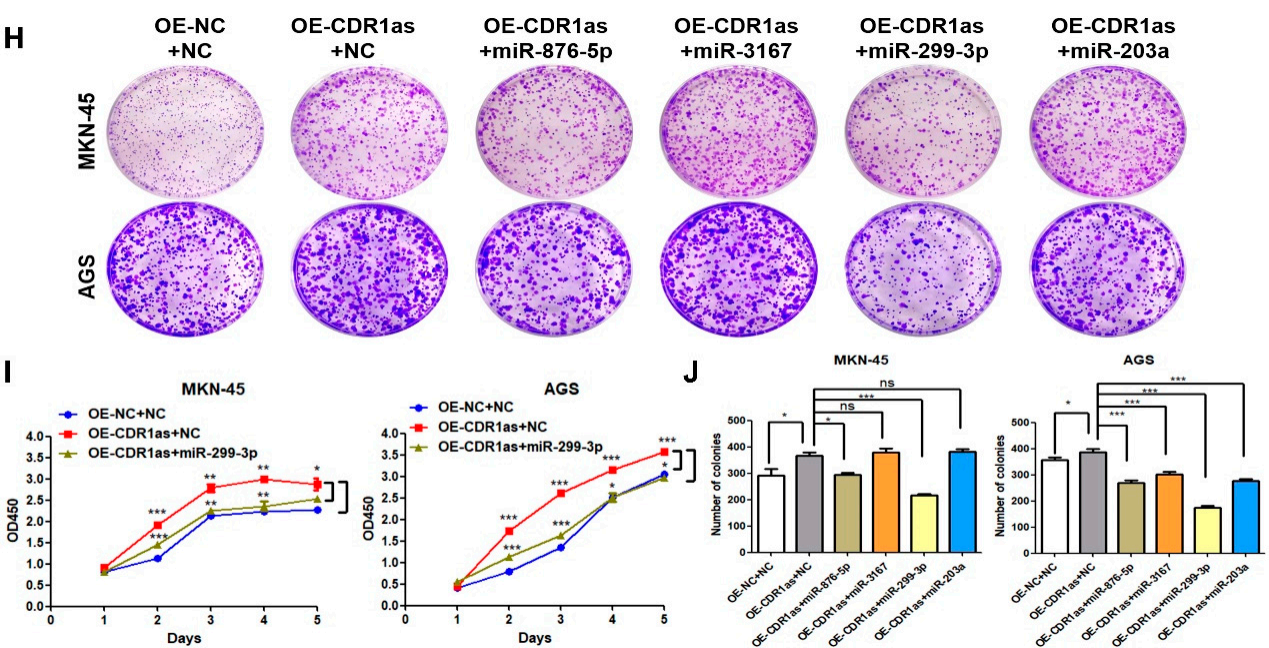
CDR1as acting as a miRNA sponge for miR-299-3p. (**A**) RNA FISH analysis for CDR1as expression in HGC-27 cells; scale bar = 10 μm. (**B**) RT-qPCR analysis of CDR1as level in the cytoplasm and nucleus of HGC-27 and MGC-803 cells; β-actin (U3) mRNA was used as the positive control for cytoplasm (nucleus). (**C**) RIP analysis of CDR1as in MGC-803 cells using anti-AGO2 antibodies; RIP enrichment was measured by RT-qPCR; FOS and U1 are the positive controls for anti-AGO2 and anti-snRNP70 antibodies, respectively. (**D**,**E**) Pearson correlation analysis of CDR1as and miR-7/miR-135a-5p levels. (**F**) Potential CDR1as-interacting miRNAs predicted by starBase v2.0. (**G**) Dual-luciferase reporter assays used to evaluate binding properties between CDR1as and miRNA candidates with the ratio of firefly and Renilla luciferase activities presented. (**H**–**J**) Colony formation assays and CCK8 assays performed to evaluate the proliferation ability of CDR1as-overexpressing cells reversed by miRNA candidates after co-transfection; bar graphs show the quantitative comparisons of colony numbers (* *p* < 0.05, ** *p* < 0.01, *** *p* < 0.001, ns none significance).

Previous studies reported that CDR1as can serve as a miR-7 sponge to regulate tumor progression [38]. Our results of the RNA FISH analysis indicated that CDR1as existed both in the cytoplasm and nucleus of the GC cells (Figure 3A). The cell fractionation analyses showed that CDR1as was preferentially localized in the cytoplasm of the GC cells (Figure 3B). The RIP assays revealed that CDR1as was detectable in the immunoprecipitates of miRNA-binding protein AGO2 (Figure 3C), suggesting that CDR1as could interact with miRNAs. We detected two reported CDR1as-binding miRNAs including miR-7 and miR-135a-5p in the GC tissues. Their expression levels were both upregulated while not significantly correlating with the CDR1as level in the GC tissues (Supplementary File S2, Figure S2A,B; Figure 3D,E).

The starBase v2.0 database (http://starbase.sysu.edu.cn/ (accessed on 11 August 2019)) was then used to predict new miRNA targets of CDR1as. Five miRNAs might have potential binding sites with CDR1as in addition to miR-7 (Figure 3F). According to the value of biComplex and the number of targetSites, we selected four miRNAs (miR-3167, miR-299-3p, miR-203a, miR-876-5p) for further validation. The dual-luciferase reporter assays showed that the luciferase activities of the reporter genes containing the CDR1as sequence were markedly decreased when co-transfected with these four miRNAs (Figure 3G). To further identify the key miRNA that CDR1as sponged to regulate the GC cell growth, we performed rescue experiments in the MKN-45 and AGS cells. The colony formation assays indicated that CDR1as promoted cell proliferation, while miR-876-5p and miR-299-3p, especially miR-299-3p, observably reversed this effect (Figure 3H,J). In addition, the GC cell viability could be enhanced by CDR1as, but it was attenuated by miR-299-3p (Figure 3I). These results suggested that CDR1as serves as a miR-299-3p sponge to regulate the GC growth.

### 3.3. CDR1as Upregulates TGIF1, a Target of miR-299-3p

**Figure 4:**
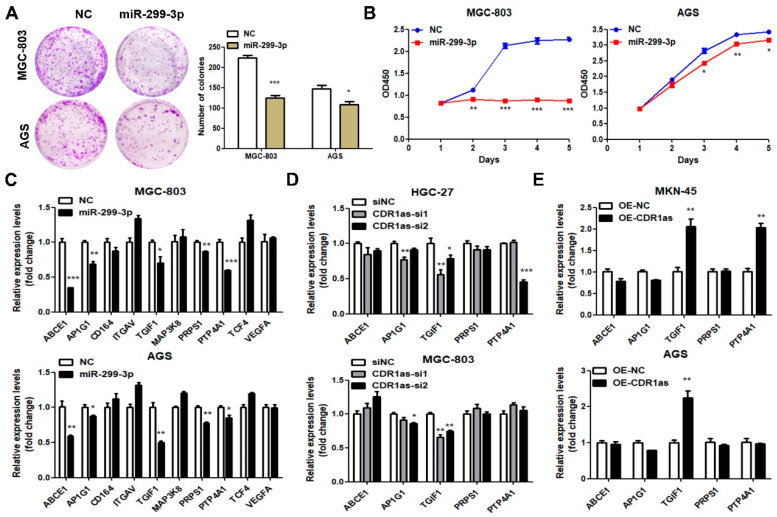

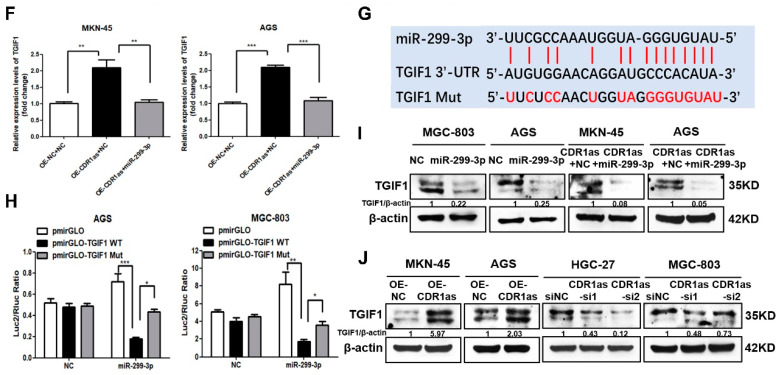
CDR1as upregulating TGIF1, a target of miR-299-3p. (**A**,**B**) Colony formation assays and CCK8 assays of MGC-803 and AGS cells transfected with miR-299-3p mimics. (**C**) RT-qPCR analysis for the expression levels of ten potential targets in MGC-803 and AGS cells after miR-299-3p mimics transfection. (**D**,**E**) RT-qPCR analysis of five miR-299-3p targets’ expression levels in GC cells with CDR1as knockdown and overexpression. (**F**) RT-qPCR analysis of TGIF1 mRNA level in CDR1as-overexpressing cells after co-transfection with miR-299-3p mimics. (**G**) Wild-type (WT) binding sequences between miR-299-3p and TGIF1 predicted by TargetScanHuman and its mutant sites (Mut). (**H**) Dual-luciferase reporter assays used to evaluate the binding ability between TGIF1 (WT or Mut 3′ UTR) and miR-299-3p with the ratio of firefly and Renilla luciferase activities presented. (**I**,**J**) Western blot analysis of TGIF1 protein level after miR-299-3p mimics transfection, CDR1as co-transfection with miR-299-3p mimics, CDR1as overexpression and knockdown (* *p* < 0.05, ** *p* < 0.01, *** *p* < 0.001). The original western blot of Figure 4I,J is in Supplementary File S3.

We next explored the effects of miR-299-3p and its downstream target in GC. The colony formation and CCK8 assays showed that miR-299-3p markedly inhibited the GC cell proliferation ability and viability (Figure 4A,B). The starBase v2.0 was applied to predict the miR-299-3p-mRNA interaction according to the coincidence degree (Supplementary File S1, Table S3). We mainly focused on the oncogenes since miR-299-3p had tumor-suppressive effects on the GC growth. Ten candidate targets including ABCE1, AP1G1, CD164, ITGAV, TGIF1, MAP3K8, PRPS1, PTP4A1, TCF4 and VEGFA were selected. After the transfection with miR-299-3p mimics in the MGC-803 and AGS cells, five genes including ABCE1, AP1G1, TGIF1, PRPS1 and PTP4A1 were significantly downregulated (Figure 4C), suggesting that these genes might be potential targets of miR-299-3p. To identify which gene was regulated by CDR1as when it was sponging miR-299-3p, we detected their expression levels in the GC cells after the CDR1as knockdown and overexpression. Only the TGIF1 mRNA level was decreased in the HGC-27 and MGC-803 cells with the CDR1as knockdown, while it was increased in the AGS and MKN-45 cells with the CDR1as overexpression (Figure 4D,E). Moreover, the upregulation of the TGIF1 mRNA level in the CDR1as-overexpressing AGS and MKN-45 cells was reversed by the co-transfection with miR-299-3p mimics (Figure 4F). Similar changes were also observed in the TGIF1 protein level (Figure 4I,J). Additionally, TargetScanHuman (https://www.targetscan.org/vert_71/ (accessed on 11 May 2023).) indicated that miR-299-3p could interact with the 3′ untranslated region (3′-UTR) of TGIF1 (Figure 4G; Supplementary File S2, Figure S2C). The dual-luciferase reporter assays presented that the miR-299-3p markedly decreased the luciferase activities of the reporter plasmid containing the 3′-UTR sequence of TGIF1. When the binding sequence for miR-299-3p was mutant, such an effect was suppressed (Figure 4H), suggesting that miR-299-3p regulated the TGIF1 expression by binding to its 3′-UTR. Taken together, these results revealed that TGIF1 is a downstream target of miR-299-3p and is regulated by CDR1as. 

### 3.4. Oncogenic TGIF1 Mediates the Promotive Effects of CDR1as on GC Growth 

**Figure 5:**
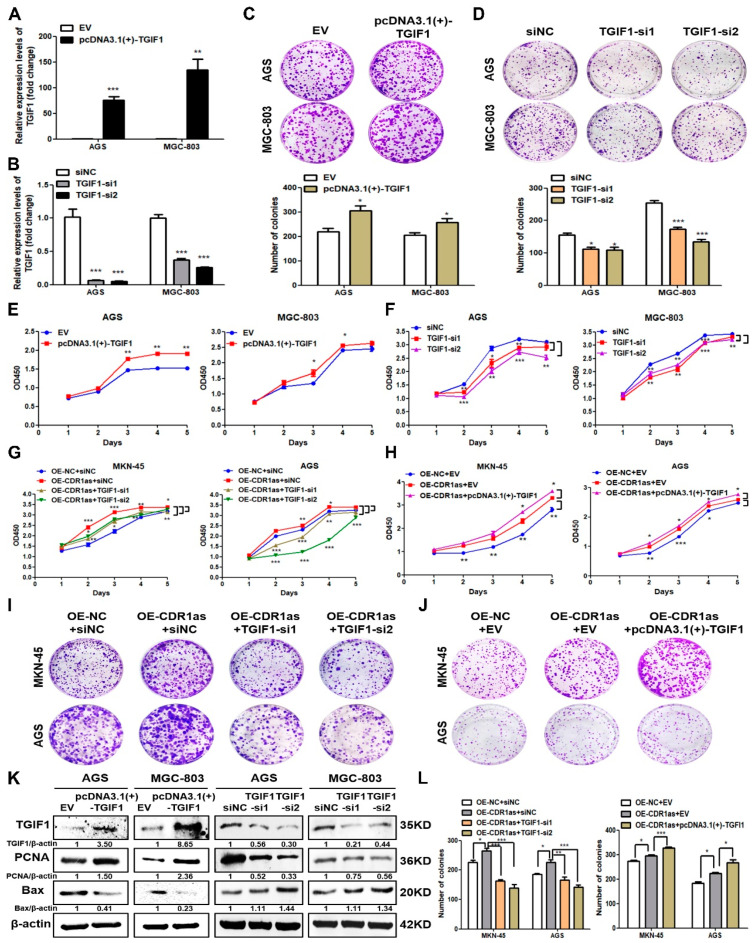
Oncogenic TGIF1 mediating the promotive effects of CDR1as on GC growth. (**A**,**B**) RT-qPCR analysis of TGIF1 mRNA level in AGS and MGC-803 cells after its overexpression and knockdown. (**C**–**F**) Colony formation assays and CCK8 assays of AGS and MGC-803 cells after TGIF1 overexpression and knockdown; bar graphs show the quantitative comparisons of colony numbers. (**G**–**J**) CCK8 and colony formation assays of CDR1as-overexpressing GC cells co-transfected with TGIF1-overexpressing vectors or TGIF1 siRNAs. (**K**) Western blot analysis for the TGIF1, PCNA and Bax protein levels in GC cells with overexpression and knockdown of TGIF1. (**L**) Bar graphs showing the quantitative comparisons of colony numbers (* *p <* 0.05, ** *p <* 0.01, *** *p <* 0.001). The original western blot of Figure 5K is in Supplementary File S3.

We next evaluated the biological role of TGIF1 in the GC cell growth. TGIF1 was overexpressed in the GC cells, and the overexpression efficiency was verified by the RT-qPCR and the Western blot analysis (Figure 5A,K). The colony formation and CCK8 assays showed that the TGIF1 overexpression promoted the proliferation of the GC cells (Figure 5C) and enhanced their viability (Figure 5E). We also knocked down the TGIF1 level in the AGS and MGC-803 cells with two different siRNAs (Figure 5B,K). The TGIF1 knockdown markedly suppressed the proliferation and viability ability of the GC cells (Figure 5D,F). In addition, the Western blot indicated that TGIF1 increased the level of the proliferation-related protein PCNA and decreased that of the apoptosis-related protein Bax in the GC cells (Figure 5K). Conversely, when TGIF1 was knocked down in the AGS and MGC-803 cells, the PCNA protein level was decreased while that of Bax was increased (Figure 5K). These results indicated that TGIF1 has oncogenic effects on the GC growth. Furthermore, we observed that the TGIF1 protein level was increased in the CDR1as-overexpressing group of subcutaneous tumor tissues (Supplementary File S2, Figure S3A). Meanwhile, the expression of PCNA was increased while that of Bax was decreased. When the TGIF1 level was knocked down in the CDR1as-overexpressing cells, the CDR1as effects on cell viability and proliferation were significantly inhibited or even reversed (Figure 5G,I,L). When we overexpressed TGIF1 in the CDR1as-overexpressing GC cells, the promotive effects of CDR1as on cell viability and proliferation ability were significantly enhanced (Figure 5H,J,L). Thus, the oncogenic TGIF1 was an important regulator mediating the promotive effects of CDR1as on the GC cell growth.

### 3.5. HuR Interacts with CDR1as and Upregulates Its Level and TGIF1

**Figure 6:**
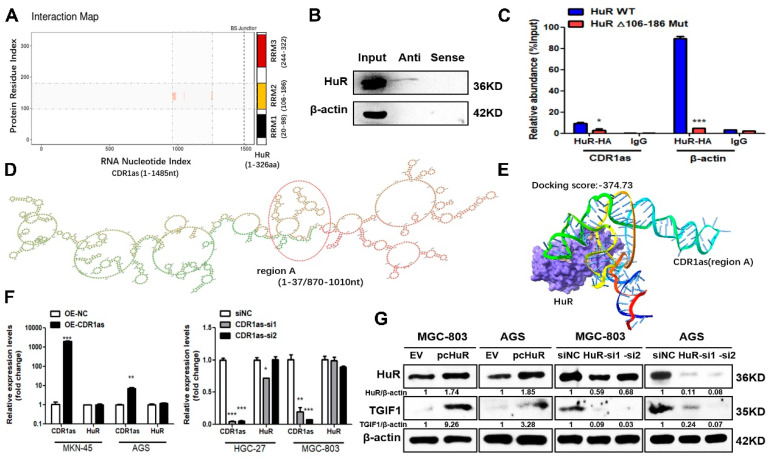

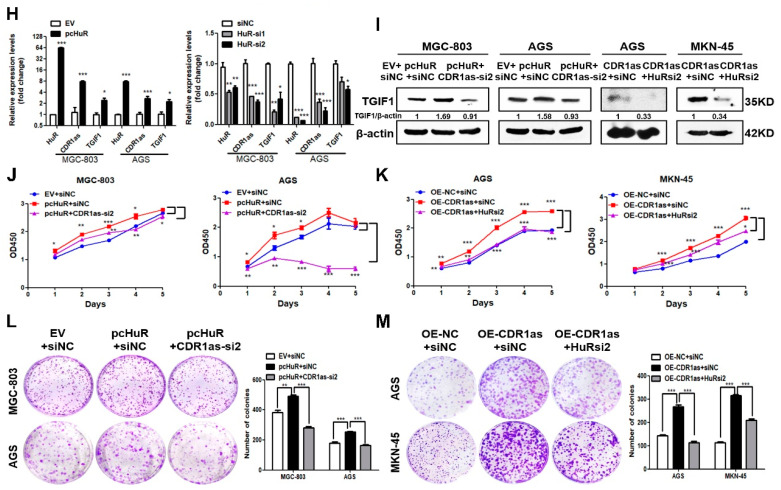
HuR interacting with CDR1as and upregulating the CDR1as and TGIF1 levels. (**A**) CDR1as-HuR interaction map predicted by catRAPID algorithm. (**B**) RNA pull-down assays using biotin-labeled antisense (Anti) and sense probes for CDR1as. (**C**) RIP assays of CDR1as-overexpresssing MKN-45 cells after transfection with HA-tagged full-length (WT) and RRM2 deletion mutant (△106–186 Mut) of HuR; β-actin is the positive control for the anti-HuR antibody. (**D**) Optimal secondary structure of CDR1as predicted by the RNAfold server; the interaction region of CDR1as with HuR is marked as region A. (**E**) Predicted 3D model of CDR1as (region A) and HuR interaction via HDOCK. (**F**) RT-qPCR analysis of HuR level after CDR1as overexpression and knockdown. (**G**,**H**) Western blot and RT-qPCR analyses of CDR1as and TGIF1 levels after HuR overexpression and knockdown. (**I**) TGIF1 protein level in HuR-overexpressing GC cells with CDR1as knockdown and in CDR1as-overexpressing GC cells with HuR knockdown. (**J**,**L**) CCK8 assays and colony formation assays of HuR-overexpressing GC cells co-transfected with the CDR1as siRNA. (**K**,**M**) CCK8 assays and colony formation assays of CDR1as-overexpressing GC cells with HuR knockdown; the bar graph shows the quantitative comparisons of colony numbers (* *p <* 0.05, ** *p <* 0.01, *** *p <* 0.001). The original western blot of Figure 6B,G,I is in Supplementary File S3.

HuR has been reported to be an important circRNA-interacting protein. The catRAPID algorithm (http://service.tartaglialab.com/page/catrapid_group (accessed on 2 April 2023)) showed that the RNA recognition motif RRM2 of HuR could interact with 1000nt downstream of the CDR1as back-spliced site (Figure 6A). We then used the in silico molecular docking approach to test the interaction ability between them. The optimal secondary structure of CDR1as was predicted by RNAfold WebServer (△G = −196.20 kcal/mol) (Figure 6D), and the potential interacting region A was then separated (1–37nt/870–1010nt) for a docking analysis based on the RNA stem-loop. The docking score was −374.73 (Figure 6E), implying the relatively strong interaction between HuR and CDR1as. To further verify this result, we applied the biotin-labeled probes to pull down CDR1as and HuR was found to exist in the precipitates of CDR1as (Figure 6B). Meanwhile, RIP assays were performed in the CDR1as-overexpressing MKN-45 cells co-transfected with HA-tagged full-length HuR vector or its RRM2 deletion mutant. The results showed that CDR1as was abundant in the immunoprecipitates of HuR protein but decreased markedly in that of the RRM2 deletion mutant (Figure 6C), suggesting that the RRM2 domain was crucial for the HuR interaction with CDR1as. 

To explore whether CDR1as could affect the HuR expression, we detected the mRNA and protein levels of HuR after the CDR1as overexpression and knockdown. We observed that CDR1as was unable to change the HuR expression level (Figure 6F; Supplementary File S2, Figure S3B). However, the CDR1as level was significantly upregulated with the HuR overexpression and downregulated after the HuR knockdown (Figure 6H). Also, the HuR overexpression and knockdown positively affected the protein and mRNA levels of the CDR1as downstream target TGIF1 (Figure 6G,H). The overexpression of HuR exhibited promotive effects on the GC cell viability and proliferation ability. However, when we knocked down the CDR1as level in the HuR-overexpressing GC cells, these effects were markedly reversed (Figure 6J,L). The level of the downstream target TGIF1 also decreased (Figure 6I). Additionally, the knockdown of HuR in the CDR1as-overexpressing GC cells significantly suppressed the promotive effects of CDR1as on the GC cell viability and proliferation ability (Figure 6K,M) and downregulated the TGIF1 level (Figure 6I). These results suggested that HuR positively regulates CDR1as and the level of its downstream TGIF1, and that such interaction is crucial for their biological functions.

## 4. Discussion

Previous studies have shown that CDR1as has important regulatory effects on tumor progression. It acts as an oncogene in colorectal cancer (CRC), hepatocellular carcinoma and BRC and as a tumor suppressor in OC and melanoma [23]. In this study, we found that CDR1as has oncogenic effects on the GC growth through the gain- and loss-of-function studies in the GC cells and subcutaneous xenograft tumor models. The cell proliferation-related and anti-apoptosis proteins were upregulated, while pro-apoptosis proteins were downregulated by CDR1as. The rate of the apoptotic GC cells was also negatively regulated. Moreover, CDR1as has many conserved binding sites for miR-7 and usually acts as a miR-7 sponge. For example, CDR1as acts as an oncogene in OS by targeting miR-7 and upregulating the EGFR and CCNE1 expression [25]. Silencing CDR1as inhibits the CRC progression by targeting miR-7 and downregulating the EGFR and IGF-1R expression [39]. In this study, CDR1as was found to sponge a new miRNA miR-299-3p in the GC growth. The bioinformatics analysis combined with the RIP assays and dual-luciferase reporter gene assays verified their binding ability. The cell function assays and rescue experiments confirmed that miR-299-3p exerted suppressive effects and could reverse the promotive role of CDR1as in the GC cell growth. MiR-299-3p has been previously reported to act as a tumor suppressor and induce cell apoptosis [40]. Here, we reported its suppressive effects on GC and identified CDR1as as a sponge of miR-299-3p.

TGF-β-induced factor homeobox 1 (TGIF1) is a transcriptional repressor. It can interfere with retinoid X receptor (RXR) binding to DNA or recruit corepressors including histone deacetylases (HDACs) to TGF-β signaling to regulate various biological processes [41]. Previous studies suggested that TGIF1 acts as an oncogene in tumorigenesis. In NSCLC, it promotes the growth and migration of cancer cells by activating the β-catenin/TCF signaling pathway [42]. In BRC, the TGIF1 upregulation is associated with poor prognosis and promotes the Wnt1-driven tumor progression [43]. In this study, TGIF1 also exerted an oncogenic role in the GC growth with the regulation of PCNA and Bax. Moreover, TGIF1 was identified as a new downstream target gene of miR-299-3p with bioinformatic prediction combined with the dual-luciferase reporter gene assays, RT-qPCR and Western blot analyses. Meanwhile, we found that TGIF1 mediated the CDR1as effects on the GC growth. CDR1as could upregulate the TGIF1 level in vitro and in vivo. They were both able to positively regulate PCNA and negatively regulate Bax. In addition, the CDR1as promotive effects on the GC cell growth were inhibited, and the TGIF1 expression was downregulated by rescue with miR-299-3p. When the TGIF1 level was knocked down or overexpressed, the promotive effects of CDR1as were correspondingly inhibited or enhanced, revealing that TGIF1 is a crucial effector of CDR1as in the GC growth.

HuR is a member of the embryonic lethal abnormal visual protein (ELAV) family which is responsible for the stabilization and translation of target mRNAs through binding to poly-U elements or AU-rich elements (AREs) in the 3′-UTR with its three RNA recognition motifs (RRMs). As an RNA binding protein (RBP), HuR enhances proto-oncogenes, cytokines and growth factors and exerts promotive effects in multiple cancers [44]. It has been recently reported to regulate tumor progression via interacting with many different circRNAs. In CC, oncogenic circTICRR interacts with HuR via binding to F287/F289 in the RRM3 domain to stabilize GLUD1 mRNA [45]. CircRHOBTB3 binds to and degrades HuR to reduce PTBP1 mRNA stability which suppresses the metastasis of CRC [46]. In this study, bioinformatic tools combined with the RIP and RNA pull-down assays proved that HuR interacted with CDR1as via the RRM2 domain. However, CDR1as was not observed to affect the HuR level. Instead, we reported that HuR binds to CDR1as and positively regulates its level and the effects on the GC growth here, which is different from the findings of the previous studies. Considering that CDR1as could be repressed by miR-671 via AGO2-slicer activity [18], we supposed that such interaction might increase the CDR1as stability. Additionally, we found that the interaction between HuR and CDR1as was crucial for their roles in the GC progression since the knockdown of CDR1as reversed the promotive role of HuR, while the decrease in HuR suppressed the CDR1as effects.

Many small molecular drugs and monoclonal antibodies targeting EGFR, VEGF or HER2 have been developed for targeted treatment for GC. However, due to the limitation of a single therapeutic target, their efficiency is still unsatisfactory [47]. Since CDR1as acts as multiple miRNA sponges including miR-7, miR-423-5p [48], miR-641 [49] and miR-299-3p to regulate various tumor-related proteins, it could be a more effective tumor therapeutic target. Targeted silencing of CDR1as might be an effective strategy here. Meanwhile, combining it with the HuR knockdown or knockout might enhance the therapeutic efficacy since it functions as a positive regulator of CDR1as. A specific siRNA, shRNA vector or CRISPR/Cas9 system combined with exosome-mediated delivery might knock down or knock out the target circRNA or protein more efficiently [50,51,52]. However, the real efficacy and safety need to be further validated. 

## 5. Conclusions

In summary, our study identified CDR1as as an oncogene in the GC progression. We demonstrated that CDR1as promotes the GC cell proliferation while suppressing their apoptosis in vitro and in vivo. Mechanically, CDR1as sponges the new target miR-299-3p to attenuate its suppression of downstream mRNA TGIF1, which in turn upregulates TGIF1 and PCNA while downregulating Bax. HuR could directly bind to CDR1as to enhance its expression level and oncogenic effects in the CDR1as/miR-299-3p/TGIF1 axis. Our study uncovered a novel mechanism of the GC progression, which expands the understanding of the CDR1as role in the GC pathogenesis and provides a promising therapeutic target for the GC treatment.

## Data Availability

All data supporting the findings of this study are available within the article, its Supplementary Files and from the corresponding author upon reasonable request.

## Supplementary Materials

The following supporting information can be downloaded at https://www.mdpi.com/article/10.3390/cancers15235556/s1. Supplementary File S1: Table S1—The sequences of gene-specific primers for RT-qPCR. Table S2—The sequences of siRNAs for gene knockdown. Table S3—Potential target mRNAs of miR-299-3p predicted via starBase v2.0. Supplementary File S2: Figure S1—CDR1as promoting GC cell growth. (A) GFP intensities in CDR1as-overexpressing GC cells and corresponding control cells. (B–D) Colony formation assays and CCK8 assays of GC cells after CDR1as overexpression and knockdown. (E) RT-qPCR analysis of CDR1as level in CDR1as-overexpressing cells and control cells. (F) Immunostaining of Bax expression of subcutaneous tumor tissues in OE-NC and OE-CDR1as groups. Figure S2—CDR1as acting as a miR-299-3p sponge. (A,B) The expression levels of miR-7 and miR-135a-5p in GC and GCN tissues with RT-qPCR analysis. (C) Potential miR-299-3p and TGIF1 mRNA interaction sites predicted by TargetScanHuman. Figure S3—CDR1as upregulating TGIF1 expression. (A) Western blot analysis of TGIF1, PCNA and Bax levels in subcutaneous tumor tissues with CDR1as overexpression. (B) Western blot analysis of HuR protein level after CDR1as overexpression and knockdown. Supplementary File S3: The original western blot of figures.

## Abbreviations

GC: gastric cancer; circRNAs: circular RNAs; siRNAs: small interfering RNAs; RT-qPCR: reverse transcription–quantitative polymerase chain reaction; GFP: green fluorescent protein; CDS: coding sequence; PI: propidium iodide; FISH: fluorescent in situ hybridization; SCC: saline sodium citrate; RIP: RNA immunoprecipitation; FBS: fetal bovine serum; IHC: immunohistochemistry; SABC: streptavidin–biotin complex; BSA: bovine serum albumin; DAB: diaminobenzidine; TGIF1: TGF-β-induced factor homeobox 1; RXR: retinoid X receptor; HDACs: histone deacetylases; ELAV: embryonic lethal abnormal visual protein; HuR: human antigen R; AREs: AU-rich elements; 3′-UTR: 3′-untranslated region; RRMs: RNA recognition motifs; RBP: RNA binding protein; NSCLC: non-small cell lung cancer; OS: osteosarcoma; OC: ovarian cancer; CRC: colorectal cancer; CC: cervical cancer; BRC: breast cancer.
