# Supplementary material for: Circular RNA CDR1as Mediated by Human Antigen R (HuR) Promotes Gastric Cancer Growth via miR-299-3p/TGIF1 Axis
Source: Cancers (Basel). 2023 Nov 23;15(23):5556. doi: 10.3390/cancers15235556 (PMC10705315; doi:10.3390/cancers15235556)
Supplement: Supplementary file 1 [file cancers-15-05556-s001.zip › Supplementary file S1.pdf]

Table S1 The sequences of gene-specific primers for RT-qPCR

Gene	Forward primer	Reverse primer
CDR1as	ACGTCTCCAGTGTGCTGA	CTTGACACAGGTGCCATC
β-actin	CTCAGGAGGAGCAATGATCT	GACCTGTACGCCAACACAGT
ABCE1	CAGCCTTTGTTGTGGAACATGA	ATTCGTGGCCTATAGTTGTTTGGA
AP1G1	TGCAATCCGGTCATCTTTTAGAG	AACTGTCCAAAGTGAGCAGGG
CD164	GTGCTGTCCGCGGACAAGAAC	TGTGAACAATAGCTCTCATC
ITGAV	AAGCTGAGCTCATCGTTTCC	GCACAGGAAAGTCTTGCTAAGG
TGIF1	GAACACAGATACAACGCCTATCC	CGTTGATGAACCAGTTACAGACC
MAPK8	ATGGAGTACATGAGCACTGGA	GCTGGCTCTTCACTTGCATAAAG
PRPS1	GGCTGACACTTGTGGCACAATC	GATGCGAGAAATAGCAGGACCG
PTP4A1	TGCTGTTCATTGCGTTGCAG	CCACGCCGCTTTTGTCTTATG
TCF4	CCACAGCTCTGACCGTCAAT	TGTTGGTGTGACTATGGCCG
VEGFA	GGGCAGAATCATCACGAAGT	TGGTGATGTTGGACTCCTCA
HuR	ATCGTCAACTACCTCCCTCA	GTGTCCTGCTACTTTATCCC

Table S2 The sequences of siRNAs for gene knockdown

siRNA	sense	antisense
NC	UUCUCCGAACGUGUCACGUTT	ACGUGACACGUUCGGAGAATT
CDR1as-si1	UCUGCAAUAUCCAGGGUUUTT	AAACCCUGGAUAUUGCAGATT
CDR1as-si2	UAUCCAGGGUUUCCGAUGGTT	CCAUCGGAAACCCUGGAUATT
TGIF1-si1	GGACAUUCCCUUGGACCUUTT	AAGGUCCAAGGGAAUGUCCTT
TGIF1-si2	GCACCGUUACAAUGCCUAUTT	AUAGGCAUUGUAACGGUGCTT
HuR-si1	GGUUUGGGCGGAUCAUCAATT	UUGAUGAUCCGCCCAAACCTT
HuR-si2	GAACGAAUUUGAUCGUCAATT	UUGACGAUCAAAUUCGUUCTT

Table S3 Potential target mRNAs of miR-299-3p predicted via starBase v2.0

miRNAname	geneID	geneName	PITA	RNA22	miRmap	microT	miRanda	PicTar	TargetScan	**coincidence number**
hsa-miR-299-3p	ENSG00000175582	RAB6A	1	0	1	1	1	1	1	6
hsa-miR-299-3p	ENSG00000010244	ZNF207	1	0	1	1	1	1	1	6
hsa-miR-299-3p	ENSG00000108848	LUC7L3	1	0	1	1	1	1	1	6
hsa-miR-299-3p	ENSG00000196628	TCF4	1	0	1	1	1	1	1	6
hsa-miR-299-3p	ENSG00000138448	ITGAV	1	0	1	1	1	1	1	6
hsa-miR-299-3p	ENSG00000124201	ZNFX1	1	0	1	1	1	1	1	6
hsa-miR-299-3p	ENSG00000198900	TOP1	1	0	1	1	1	1	1	6
hsa-miR-299-3p	ENSG00000175040	CHST2	1	0	1	1	1	1	1	6
hsa-miR-299-3p	ENSG00000087274	ADD1	1	0	1	1	1	1	1	6
hsa-miR-299-3p	ENSG00000164163	ABCE1	1	0	1	1	1	1	1	6
hsa-miR-299-3p	ENSG00000111962	UST	1	1	1	1	1	0	1	6
hsa-miR-299-3p	ENSG00000186591	UBE2H	1	0	1	1	1	1	1	6

hsa-miR-299-3p	ENSG00000105866	SP4	1	0	1	1	1	1	1	6
hsa-miR-299-3p	ENSG00000082258	CCNT2	1	0	1	1	1	1	1	6
hsa-miR-299-3p	ENSG00000107968	MAP3K8	1	0	0	1	1	1	1	5
hsa-miR-299-3p	ENSG00000171798	KNDC1	1	0	1	1	1	0	1	5
hsa-miR-299-3p	ENSG00000073614	KDM5A	1	0	1	1	1	0	1	5
hsa-miR-299-3p	ENSG00000051825	MPHOSPH9	1	0	0	1	1	1	1	5
hsa-miR-299-3p	ENSG00000103647	CORO2B	1	1	1	1	1	0	0	5
hsa-miR-299-3p	ENSG00000157766	ACAN	1	1	1	0	1	0	1	5
hsa-miR-299-3p	ENSG00000069345	DNAJA2	1	0	1	1	1	0	1	5
hsa-miR-299-3p	ENSG00000166747	AP1G1	1	0	1	1	1	1	0	5
hsa-miR-299-3p	ENSG00000108375	RNF43	1	0	1	0	1	1	1	5
hsa-miR-299-3p	ENSG00000177426	TGIF1	1	0	1	1	1	0	1	5
hsa-miR-299-3p	ENSG00000136536	7-Mar	1	0	1	1	1	0	1	5
hsa-miR-299-3p	ENSG00000116095	PLEKHA3	1	0	0	1	1	1	1	5
hsa-miR-299-3p	ENSG00000159086	PAXBP1	1	0	1	0	1	1	1	5
hsa-miR-299-3p	ENSG00000135535	CD164	1	0	1	1	1	1	0	5
hsa-miR-299-3p	ENSG00000112715	VEGFA	1	0	1	0	1	1	1	5
hsa-miR-299-3p	ENSG00000112245	PTP4A1	1	0	1	1	1	0	1	5
hsa-miR-299-3p	ENSG00000157212	PAXIP1	1	0	1	1	1	1	0	5
hsa-miR-299-3p	ENSG00000257923	CUX1	1	0	1	0	1	1	1	5
hsa-miR-299-3p	ENSG00000147224	PRPS1	1	0	1	1	1	0	1	5
hsa-miR-299-3p	ENSG00000205189	ZBTB10	1	0	1	1	1	0	1	5
