# Supplementary material for: Circular RNA CDR1as Mediated by Human Antigen R (HuR) Promotes Gastric Cancer Growth via miR-299-3p/TGIF1 Axis
Source: Cancers (Basel). 2023 Nov 23;15(23):5556. doi: 10.3390/cancers15235556 (PMC10705315; doi:10.3390/cancers15235556)
Supplement: Supplementary file 1 [file cancers-15-05556-s001.zip › Supplementary file S2.pdf]

Supplementary results

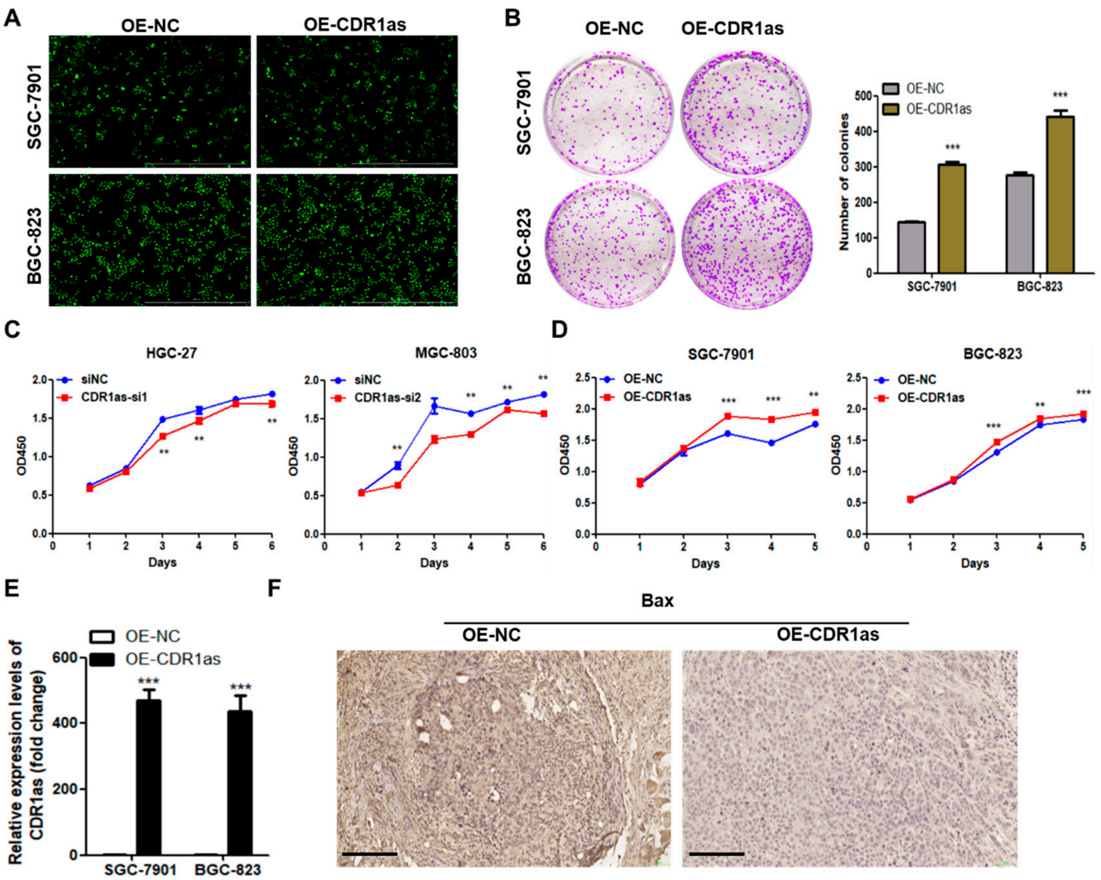

Figure S1 CDR1as promotes GC cell growth. **A** GFP intensities in CDR1as-overexpressing GC cells and corresponding control cells. The scale bar indicates 1000 μm. **B-D** Colony formation assays and CCK8 assays of GC cells after CDR1as overexpression and knockdown. **E** RT-qPCR analysis of CDR1as level in CDR1as-overexpressing cells and control cells. **F** immunostaining of Bax expression of subcutaneous tumor tissues in OE-NC and OE-CDR1as groups. The scale bar indicates 100 μm. (**p<0.01, ***p<0.001)

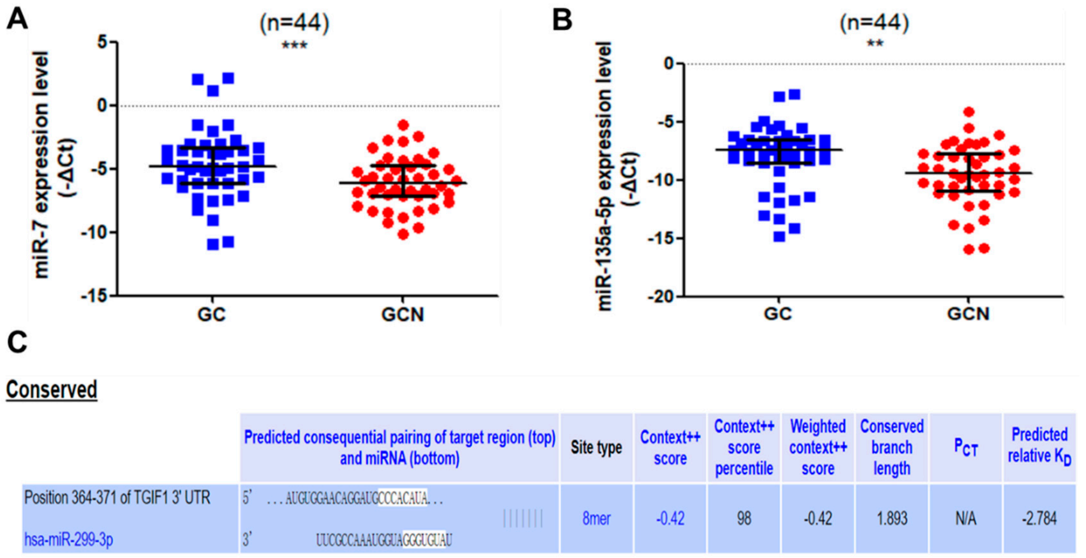

	Predicted consequential pairing of target region (top) and miRNA (bottom)	Site type	Context++ score	Context++ score percentile	Weighted context++ score	Conserved branch length	P_{CT}	Predicted relative K_D
Position 364-371 of TGIF1 3' UTR<br>hsa-miR-299-3p	5' ...AUGUGGAACAGGAUGCCCACAUA...<br>||||||| 3' UUCGCCAAAUGGUAGGGUGUAU	8mer	-0.42	98	-0.42	1.893	N/A	-2.784

Figure S2 CDR1as acts as a miR-299-3p sponge. **A, B** The expression levels of miR-7 and miR-135a-5p in GC and GCN tissues with RT-qPCR analysis. GCN represents matched noncancerous tissues. **C** Potential interacting sites between miR-299-3p and TGIF1 mRNA predicted by TargetScanHuman. (***p*<0.01, ****p*<0.001)

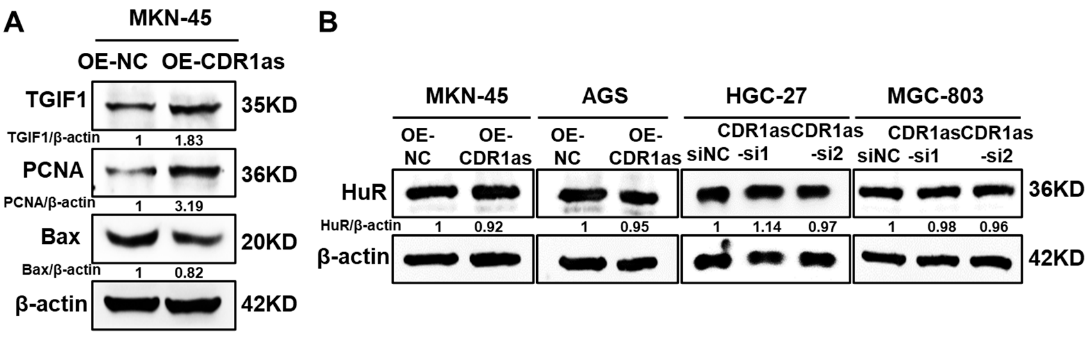

Figure S3 CDR1as upregulates TGIF1 expression. **A** Western blot analysis of TGIF1, PCNA and Bax levels in subcutaneous tumor tissues with CDR1as overexpression. **B** Western blot analysis of HuR protein level after CDR1as overexpression and knockdown.
